# Pharmacists’ Perceived Knowledge of Kratom and Hemp-Derived Cannabinoids Before and after Education

**DOI:** 10.1177/29768357261484353

**Published:** 2026-08-27

**Authors:** Kirk E. Evoy, Nina Vadiei, Diego Sierra

**Affiliations:** 1 College of Pharmacy, 12330 The University of Texas at Austin, San Antonio, TX, USA; 2 Long School of Medicine, The University of Texas at San Antonio, San Antonio, TX, USA; 3 Department of Pharmacy, University Health, San Antonio, TX, USA; 4 Department of Pharmacy, San Antonio State Hospital, San Antonio, TX, USA

**Keywords:** kratom, hemp-derived cannabinoids, pharmacist, THC, delta-8

## Abstract

**Introduction:**

Kratom and hemp-derived cannabinoid use has increased in the United States.

**Objective:**

Assess pharmacists’ perceived knowledge of kratom and hemp-derived cannabinoids and whether it improved following continuing education.

**Methods:**

This cross-sectional survey was administered to participants immediately before and after attending a live, one-hour continuing education presentation regarding the pharmacology, uses, risks, and legality of kratom and hemp-derived cannabinoids. The novel, investigator-designed, literature-informed survey assessed the frequency with which they were asked about kratom and hemp-derived cannabinoids in practice, perceived knowledge of the uses, risks, and legality of these substances, and participant’s perceived self-efficacy regarding counseling patients about them. The post-education survey also contained questions regarding perceived training value. Any pharmacist who attended the presentation and acknowledged consent was eligible to participate. Non-pharmacist attendees were excluded.

**Results:**

In total, 99 participants completed the pre-education survey and 63 the post-education survey. Most were never (81.8%) asked about kratom, while about half (48.5%) were never asked about hemp-derived cannabinoids, with 45.5% asked twice per month or less. Prior to education, most pharmacists did not feel confident discussing common uses, risks, or legal/regulatory status of kratom or hemp-derived cannabinoids and had limited understanding of distinctions between cannabis-related terms. Median pre-education scores ranged from 1-2 (out of 5) across all items. Following education, perceived knowledge and self-efficacy improved across all five items (all p<0.0001), each with post-education medians of 4.

**Conclusion:**

This exploratory study indicated that Texas pharmacists’ perceived knowledge of kratom and hemp-derived cannabinoids was low, but significantly improved following education on the topic. It also indicated they are not often asked about these substances in day-to-day practice, corroborating previous research that people who use these substances rarely discuss their use with their healthcare providers.

## Introduction

Kratom has been used in Asia for decades for therapeutic (e.g., to self-treat pain, substance use disorder, and mental health conditions) and recreational effects due to mild stimulant and opioid-like effects.^1,2^ Recently, semi-synthetic kratom derivatives (e.g., 7-hydroxymitragynine) that function as potent opioid agonists have entered the United States (US) market, posing considerable public health risks, including potential overdose and dependence risks.^3^ Despite the growing risks conferred by these highly potent kratom derivatives, there are no approved treatments for kratom use disorder, though limited evidence supports the potential use of opioid agonist therapy (e.g., buprenorphine) in severe cases.^3^

Following 2018 legislation legalizing hemp production and sale, several hemp-derived cannabinoids (e.g., delta-8, delta-10, tetrahydrocannabinolic acid [THCA]) entered the US market.^4^ These semi-synthetic products function similarly to marijuana and are used for similar purposes, particularly in states that have not legalized cannabis. However, limited regulation, quality control, and research regarding these substances likely confer greater risks than the highly-regulated cannabis sold in states with legalized dispensing.^4,5^

With the number of US smoke shops greatly increasing in recent years, numerous, largely unregulated kratom and hemp-derived cannabinoid products have entered the US market and their use has increased considerably.^6^ Lifetime US kratom use prevalence has been estimated at 1-9%,^7-10^ while delta-8 (the most studied hemp-derived cannabinoid) lifetime use prevalence has been estimated at 7.7%.^11^

As pharmacists are likely to encounter more patients using these substances, it is important that they can provide evidence-based counseling and understand their health implications when treating patients using them. This study was conducted to assess how often pharmacists are asked about kratom and hemp-derived cannabinoids in practice, their perceived knowledge of the substances, and whether it improved after receiving education.

## Methods

This cross-sectional survey was administered from 9/28/2025-4/25/2026 to participants attending one of two live, one-hour continuing education presentations regarding kratom and hemp-derived cannabinoids in Austin, and Galveston, Texas. One was predominantly attended by participants from Austin and San Antonio, Texas, while the other was included participants from across the state. The total number of attendees was not captured, so response rates were unavailable. The presentation discussed pharmacology, uses, risks, and legality of kratom and hemp-derived cannabinoids. The electronic survey was administered immediately before and immediately after the presentation via Qualtrics® (Provo, UT). The investigator-designed, literature-informed,^1,3,4,12,13^ survey developed for this study included multiple choice and Likert-type questions assessing demographics, frequency with which they were asked about kratom and hemp-derived cannabinoids in practice, perceived knowledge of the uses, risks, and legality of these substances, and participant’s perceived self-efficacy regarding counseling patients about them. The post-education survey contained the same questions as the pre-education survey plus two additional questions regarding perceived value of the training. The survey was piloted by 11 (11% of total sample) healthcare professionals prior to the conference. Participation was voluntary and anonymous. Consent information was provided prior to starting the survey. Inclusion criteria included any pharmacist (self-reported) who attended the presentation and acknowledged consent, while exclusion criteria included any non-pharmacist attendees. No compensation was provided for participation. The study was deemed non-human research on 12/5/2024 by the University of Texas Health San Antonio Institutional Review Board (Protocol 00001180).

Statistical analyses were conducted using Microsoft Excel® (Redmond, WA) and JMP (SAS Corp., Cary, NC). Demographic characteristics were summarized descriptively and compared between pre- and post-education using chi-square tests. Pre- and post-education survey responses were treated as independent samples; between-group differences in perceived knowledge and self-efficacy were assessed using the Wilcoxon rank-sum test. A p-value of <0.05 indicated significance.

## Results

### Respondent Demographics and Experience With Kratom and Hemp-derived Cannabinoids

In total, 99 participants completed the pre-education survey and 63 the post-education survey. Participants were predominantly female, practiced in community or hospital pharmacy, and had been practicing for ≥20 years (Table 1). Most were never (81.8%) asked about kratom, while a lower proportion were asked about it less than once per month (10.1%), once or twice per month (6.1%) or more than twice per month (2%). About half (48.5%) were never asked about hemp-derived cannabinoids, 25.3% were asked about them less than once per month, 20.2% once or twice a month, and 6.1% more than twice per month.

**Table 1.** Participant Demographics

​	Pre-education n (%)^^^	Post-education n (%)^^^	P-value^*^
Gender:	​	​	0.482
• Female	56 (56.6)	40 (63.5)	
• Male	40 (40.4)	21 (33.3)	
• Other/did not disclose	3 (3)	2 (3.2)	
How many years have you been practicing?	​	​	0.636
• 0-10 years	10 (10.3)	8 (13.1)	
• 11-20 years	15 (15.5)	12 (19.7)	
• 20+ years	72 (74.2)	41 (67.2)	
Practice setting:	​	​	0.927
• Inpatient hospital or acute care facility	24 (24.7)	15 (24.6)	
• Outpatient clinic	6 (6.2)	5 (8.2)	
• Community pharmacy	37 (38.1)	23 (37.7)	
• Management/administration	11 (11.3)	4 (6.6)	
• Academia	1 (1)	1 (1.6)	
• Other	18 (18.6)	13 (21.3)	
Practice specialty:	​	​	0.906
• Community pharmacy	37 (38.1)	24 (39.3)	
• Internal medicine (inpatient)	16 (16.5)	8 (13.1)	
• Pediatrics	2 (2.1)	2 (3.3)	
• Primary care/internal medicine (outpatient)	7 (7.2)	7 (11.5)	
• Psychiatry/substance use disorder	1 (1)	1 (1.6)	
• Other	34 (35.1)	19 (31.1)	

^^^For percentages, the denominator is total number of respondents for that specific question, as responses were not required to finish survey.

*Chi-square results for gender, practice setting, and practice specialty should be interpreted with caution as >20% of cells had expected counts <5.”

### Education Efficacy Assessment

As displayed in Table 2, prior to education, most pharmacists did not feel confident discussing common uses, risks, or legal/regulatory status of kratom or hemp-derived cannabinoids and had limited understanding of distinctions between cannabis-related terms. Median pre-education scores ranged from 1-2 (out of 5) across all items. Following education, perceived knowledge and self-efficacy improved across all five items (all p<0.0001), each with post-education medians of 4. Furthermore, 87.7% indicated that the presentation improved their ability to answer patient questions and provide education, while 96.6% agreed that healthcare professionals should receive more training on these substances.

**Table 2.** Perceived Knowledge of Kratom and Hemp-Derived Cannabinoids and Self-Efficacy Regarding Counseling Patients on These Substances

Please rate your agreement with the following statements:	Pre-education n (%)^^^	Post-education n (%)^^^	Median (IQR) pre-/Post-education	P-value
I feel confident discussing the common uses and risks of kratom.	​	​	PRE: 1 (1-2)	<0.0001
1. Strongly disagree	66 (66.7)	4 (6.3)		
2. Somewhat disagree	16 (16.2)	3 (4.8)		
3. Neither agree nor disagree	8 (8.1)	3 (4.8)	POST: 4 (4-5)	
4. Somewhat agree	5 (5.1)	38 (60.3)		
5. Strongly agree	4 (4.0)	15 (23.8)		
I feel confident discussing the common uses and risks of hemp-derived cannabinoids.	​	​	PRE: 2 (1-3)	<0.0001
1. Strongly disagree	42 (42.9)	4 (6.3)		
2. Somewhat disagree	18 (18.4)	4 (6.3)	POST: 4 (4-5)	
3. Neither agree nor disagree	17 (17.3)	3 (4.8)		
4. Somewhat agree	18 (18.4)	39 (61.9)		
5. Strongly agree	3 (3.1)	13 (20.6)		
I feel confident explaining the difference between the terms CBD, THC, hemp, and cannabis.	​	​	PRE: 2 (1-4)	<0.0001
1. Strongly disagree	36 (36.4)	2 (3.2)		
2. Somewhat disagree	27 (27.3)	4 (6.5)		
3. Neither agree nor disagree	9 (9.1)	5 (8.1)	POST: 4 (4-5)	
4. Somewhat agree	21 (21.2)	35 (56.5)		
5. Strongly agree	6 (6.1)	16 (25.8)		
I understand the legal and regulatory status of kratom products in my state and in the U.S.	​	​	PRE: 1 (1-2)	<0.0001
1. Strongly disagree	66 (66.7)	3 (4.8)		
2. Somewhat disagree	13 (13.1)	4 (6.3)		
3. Neither agree nor disagree	9 (9.1)	2 (3.2)	POST: 4 (4-5)	
4. Somewhat agree	9 (9.1)	36 (57.1)		
5. Strongly agree	2 (2)	18 (28.6)		
I understand the legal and regulatory status of hemp-derived cannabinoids in my state and the U.S.	​	​	PRE: 1 (1-2)	<0.0001
1. Strongly disagree	44 (44.4)	3 (4.8)		
2. Somewhat disagree	19 (19.2)	3 (4.8)		
3. Neither agree nor disagree	13 (13.1)	3 (4.8)	POST: 4 (4-5)	
4. Somewhat agree	20 (20.2)	37 (58.7)		
5. Strongly agree	3 (3)	17 (27.0)		
I believe this presentation improved my ability to answer patient questions and provide education regarding kratom and hemp-derived cannabinoids.^*^	​	​	-	​
1. Strongly disagree	-	4 (6.9)		-
2. Somewhat disagree	-	1 (1.7)		-
			-	
3. Neither agree nor disagree	-	2 (3.4)		-
4. Somewhat agree	-	17 (29.3)		-
5. Strongly agree	-	34 (58.6)		-

*This question was only asked in the post-education survey.

^^^For percentages, the denominator is total number of respondents for that specific question, as responses were not required to finish survey. Likert scale: 1 = Strongly Disagree, 2 = Somewhat Disagree, 3 = Neither Agree nor Disagree, 4 = Somewhat Agree, 5 = Strongly Agree. P-values derived from Wilcoxon rank-sum test (Mann-Whitney U). IQR = interquartile range.

## Discussion

Most surveyed pharmacists perceived their knowledge regarding kratom and hemp-derived cannabinoids to be low, but significantly improved following a one-hour presentation on the topic. This research built on the single previous study assessing pharmacist kratom knowledge, identifying that only 14% of Alabama pharmacists considered themselves knowledgeable about kratom and 50% had never heard of it.^12^ Given the small sample and inclusion of only Alabama pharmacists, corroborating evidence from other regions is important to assess generalizability. Furthermore, no previous research was identified assessing pharmacists’ knowledge regarding hemp-derived cannabinoids. Pharmacists have the opportunity to positively impact the health of patients using, or considering using, these substances by identifying their use during social/medication histories, providing evidence-based education regarding their risks, mitigating associated drug-drug/drug-disease interactions, guiding taper plans to minimize withdrawal, and helping manage medical conditions that may motivate their use.^14^ However, most people who use these substances rarely disclose their use to healthcare providers and are not confident in their providers’ knowledge of these substances.^15,16^ Instead they tend to rely on unreliable, non-evidence-based information (e.g., internet, friends/family, smoke shop employees, self-experimentation) to guide their use.^15^ Though most pharmacists indicated that they are not often asked about these substances, greater awareness and knowledge of these products and training regarding routinely inquiring about their use in a non-stigmatizing manner may lead to more such conversations and opportunities to positively intervene. This study indicated that even relatively brief education on these substances significantly increased pharmacists’ knowledge of kratom and hemp-derived cannabinoids and self-efficacy with regards to counseling, and almost all attendees believed that pharmacists should receive additional education on these substances.

### Limitations

The small sample size and relatively late-career, Texas-based population limit generalizability. Sample size calculations were not performed *a priori*, as the sample was limited by the number of conference attendees. Social desirability bias may also affect results. Those practicing in non-clinical roles may be less likely to be asked about these substances in their practice. Only two-thirds of respondents completed both the pre- and post-education survey, and there are inherent limitations in pre/post education assessments that may skew towards positive results. Thus, the education efficacy assessment should be viewed as exploratory and confirmed in larger, more geographically diverse samples. It may also be beneficial to assess knowledge-based questions as objective efficacy measures and to reassess knowledge gains after more time has passed since receiving education. However, the post-education data did show marked, statistically significant improvement in perceived knowledge despite the small sample.

## Conclusion

This exploratory study indicated that Texas pharmacists’ perceived knowledge of kratom and hemp-derived cannabinoids was low, but significantly improved following education on the topic. It also indicated they are not often asked about these substances in day-to-day practice, corroborating previous research that people who use these substances rarely discuss their use with their healthcare providers. Efforts to improve pharmacists’ knowledge regarding kratom and hemp-derived cannabinoids and to create a non-stigmatizing environment in which patients feel open to discuss their substance use may benefit those using or considering use of these substances.

## Supplemental Material

Supplemental Material - Pharmacists’ Perceived Knowledge of Kratom and Hemp-Derived Cannabinoids Before and after Education Supplemental Material for Pharmacists’ Perceived Knowledge of Kratom and Hemp-Derived Cannabinoids Before and after Education by Kirk E. Evoy, Nina Vadiei, Diego Sierra in Substance Use: Research and Treatment.

## Data Availability

Available upon request.*
