# Supplementary material for: Pharmacists’ Perceived Knowledge of Kratom and Hemp-Derived Cannabinoids Before and after Education
Source: Subst Use. 2026 Aug 27;20:29768357261484353. doi: 10.1177/29768357261484353 (PMC13522675; doi:10.1177/29768357261484353)
Supplement: Supplementary file 1 [file sj-pdf-1-sat-10.1177_29768357261484353.pdf]

SURVEY

For the purposes of this study, "hemp-derived THC products" refers to semi-synthetic cannabinoid products derived from hemp (e.g., delta-8, delta-9, delta-10, HHC, THCA). Kratom refers to any kratom-related product, including natural whole-leaf kratom formulations, combination kratom products (e.g., kratom + kava), or semi-synthetic derivatives (e.g., 7-hydroxymitragynine (7-OH) or mitragynine pseudoindoxyl).

Multiple choice questions:

1. How often have you had patients in your clinical practice ask about **kratom** or disclose that they use **kratom**?
    - a. Never
    - b. Rarely (less than once per month)
    - c. Sometimes (once or twice a month)
    - d. Often (2+ times per month)

2. How often have you had patients in your clinical practice ask about **hemp-derived THC** products or disclose that they use **hemp-derived THC** products?
    - a. Never
    - b. Rarely (less than once per month)
    - c. Sometimes (once or twice a month)
    - d. Often (2+ times per month)

Please rate your agreement with the following statements on a scale from 1 (Strongly Disagree) to 5 (Strongly Agree):

1. I feel confident discussing the common uses and risks of **kratom**.
2. I feel confident discussing the common uses and risks of **hemp-derived THC** products.
3. I feel confident explaining the difference between the terms CBD, THC, hemp, and cannabis.
4. I understand the legal and regulatory status of **kratom** products in my state and the U.S.
5. I understand the legal and regulatory status of **hemp-derived THC** products in my state and the U.S.

Demographics

6. What is your gender?
    - a. Male
    - b. Female
    - c. Other
7. What is your profession?
    - d. Pharmacist
    - e. Physician
    - f. Physician assistant
    - g. Nurse practitioner
    - h. Nurse
    - i. Other___________
8. How many years have you been licensed in your healthcare profession?
    - j. 0-5 years
    - k. 6-10 years

l. 11-20 years
m. 21-30 years
n. 30+ years

9. Which of the following best describes your current practice setting?
    o. Inpatient hospital or acute care facility
    p. Outpatient clinic
    q. Community pharmacy
    r. Long-term care facility
    s. Not currently in clinical practice - management/administration
    t. Not currently in clinical practice - academician
    u. Other _________
10. Which of the following best describes your current practice specialty?
    a. Primary care
    b. Internal medicine (inpatient)
    c. Pain management
    d. Psychiatry/substance use disorder
    e. Neurology
    f. Pediatrics
    g. Community pharmacy
    h. Other_________

Additional questions only included in post-education survey:

1. Do you believe healthcare professionals should have more training on substances like kratom and hemp-derived THC?
    a. Yes
    b. No
    c. Unsure

2. **Please rate your agreement with the following statements on a scale from 1 (Strongly Disagree) to 5 (Strongly Agree):** I believe this presentation improved my ability to answer patient questions and provide education regarding kratom and hemp-derived THC?
